# Mobile Phone Use Among Medical Residents: A Cross-Sectional Multicenter Survey in Saudi Arabia

**DOI:** 10.2196/mhealth.4904

**Published:** 2016-05-19

**Authors:** Amr Jamal, Mohamad-Hani Temsah, Samina A Khan, Ayman Al-Eyadhy, Cristina Koppel, Michael F Chiang

**Affiliations:** ^1^ College of Medicine Department of Family and Community Medicine King Saud University Riyadh Saudi Arabia; ^2^ School of Medicine Medical Informatics and Clinical Epidemiology (DMICE) Oregon Health & Science University Portland, OR United States; ^3^ College of Medicine Department of Paediatrics King Saud University Riyadh Saudi Arabia; ^4^ Medical Informatics and E-Learning Unit, College of Medicine Medical Education Department King Saud University Riyadh Saudi Arabia; ^5^ Department of Medicine School of Medicine Imperial College London United Kingdom; ^6^ School of Medicine Department of Ophthalmology & Medical Informatics and Clinical Epidemiology (DMICE) Oregon Health & Science University Portland, OR United States

**Keywords:** cell phones, mobile phone, telemedicine, medical education, medical residencies, educational techniques, patient care, communication methods, WhatsApp, Saudi Arabia, point of care technology

## Abstract

**Background:**

Mobile phones have great potential for medical education, as they allow health care providers and students to access resources efficiently at the precise time at the point-of-care to help in informed decision making.

**Objective:**

The objective of the study was to evaluate the prevalence of mobile phone usage among medical residents and to explore their attitudes, perceptions, and the challenges they experience when using mobile phones in academic and clinical practice.

**Methods:**

A cross-sectional survey was conducted on all 133 residents in 17 different specialties across two large academic hospitals in Riyadh, Saudi Arabia. The Web-based validated questionnaire measured mobile phone platform preferences, and their uses in general and medical practice. The perception of confidentiality and safety impact of using mobile phones for communication and accessing patient’s data was also explored, alongside challenges of use and how residents learn to use their mobile phone.

**Results:**

With a response rate of 101/133 (75.9%) and mean age of 27.8 (SD 3.0) years, we found that 100/101 (99.0%) of participants were mobile phone users with mean duration of use of 5.12 (SD 2.4) years, and a range from 1 to 12 years. There was no significant difference in use between male and female respondents. A negative linear correlation was found between age and use duration (*P*=.004). The most common operating system used by participants was the iOS platform (55/101, 54.5%), with English the most commonly used language to operate residents’ mobile phones (96/100, 96.0%) despite their native language being Arabic. For communication outside medical practice, chatting applications such as WhatsApp matched phone calls as most commonly used tools (each 88/101, 87.1%). These were also the primary tools for medical communication, but used at a lower rate (each 65/101, 64.4%). In medical practice, drug (83/101, 82.2%) and medical (80/101, 79.2%) references and medical calculation applications (61/101, 60.4%) were the most commonly used. Short battery life (48/92, 52%) was the most common technical difficulty, and distraction at least on a weekly basis (54/92, 58%) was the most likely side effect of using a mobile phone in medical practice. Practically, all participants agreed with the idea of integrating medical staff mobile phones with the hospital information system. Most residents described themselves as self-learners, while half learned from peers, and a quarter learned from the Internet. Only 7/101 (6.9%) had received formal training on the medical use of mobile phones. Over half of residents thought it was safe to discuss patients over their personal, nonencrypted email.

**Conclusions:**

Mobile phone use among medical residents has become almost universal in academic and clinical settings. Thus, academic and health care institutions should support proper utilization of these devices in medical training and point-of-care decision making, while continuing to protect patient confidentiality.

## Introduction

### Smartphones and Health Care

Over the last two decades [1], smartphones have been evolving rapidly in functionality and propagation. Smartphones combine a mobile phone with other features of personal digital assistance such as Internet browsing, email access, global positioning system navigation, touchscreen, motion sensor, wireless Internet for frequent interface/fourth generation (mobile telecommunications technology) connectivity, desktop synchronization, voice recognition, high-quality camera, large displays, as well as third party applications, commonly referred to as “apps” [2]. These functions turn the smartphone into a portable computer. They have great potential for medical education, as they allow health care providers and students to access resources efficiently at the right time at the point-of-care to support better decision making in patient care [3-7]. Faster processors, improved memory, and long-life batteries in concert with highly efficient operating systems capable of advanced functions have paved the way for apps that are beneficial for both personal and work environments.

Mobile phones in many ways are similar to personal digital assistant (PDAs), which have been studied well in health care education [4] and proven their value across a variety of apps. PDAs allowed health care providers to carry multiple references in their pocket, log clinical encounters, and tally of clinical time. Mobile phones are newer technologies with the expanded functions of cellular technology, Internet connectivity, and a wider range of specialized apps. Operating systems include, Google’s Android, Apple’s iOS, Research in Motion’s BlackBerry, and Microsoft Windows Phone platform [5]. In addition to Internet browser access, a plethora of free and paid apps are on offer in each system’s app distribution store. Here, individual users can browse and download apps as required, allowing for high personalization [8]. Mobile phones’ cellular connectivity enables them to connect to Internet resources even when wireless networks are unavailable [5], making them particularly valuable in the clinical setting.

Health care workflow is highly mobile, encompassing multiple settings of care such as outpatient clinics, inpatient wards, emergency departments, operating theaters, intensive care units (ICUs), radiology departments, laboratories, etc [6]. Consequently, working in the health care system requires extensive mobility of health care providers as well as communication and collaboration among a variety of individuals, including colleagues, multidisciplinary teams, and patients. In addition, the nature of medical resident’s work involves also continuous educational assignments and connectivity to educators and administrative teams.

Today, mobile phones serve a vital role in the practice of medicine, which ranges from patient monitoring and diagnostics to health education and communication.

### Aim of the Study

The amount of research in the use of mobile phones in medicine is rapidly growing, but there are few high-quality studies answering questions about their use and impact on medical care and education [3]. Given the rate of uptake of mobile phones, their prevalence needs to be measured frequently as figures quickly become outdated. In addition, we seek to explore attitudes, perceptions, as well as challenges faced by their users. In particular, we are interested in their effect on confidentiality and security. We also wish to shed light on how residents view the role of the institution with respect to mobile phone use and how residents learn to use their devices and overcome barriers they encounter. A better understanding of the above is the first step in maximizing the technology’s potential and limiting less desired consequences.

## Methods

### Study Design and Instrument

This is a cross-sectional survey based study using a self-administered structured questionnaire in the English language. The questionnaire included 22 validated questions distributed into the following sections: (1) general demographic information, (2) mobile phone preferences, (3) mobile phone general uses, (4) mobile phone medical-practice uses, (5) mobile phone learning/training uses, (6) communication tools/apps uses, and (7) mobile phones privacy and security issues.

### Validity

The questions were generated based on a literature review about mobile phone uses among medical professionals and students [4,5,9-12]. Then, questions were drafted using the focus group technique utilizing the authors’ personal and professional experiences. There were three experts in informatics that reviewed the questionnaire for content accuracy, validity, and reliability. A pilot study was conducted where the initial draft of the questionnaire was distributed to 30 professionals at King Khalid University Hospital (KKUH) ICU. The aim of the pilot study was to ensure the understanding and applicability of each question. Notes from pilot respondents were taken and questions were modified accordingly. A Cronbach alpha >.6 was recorded for the questionnaire during the pilot testing.

### Subjects

Invitations to participate in the survey were sent to all of the 128 active residents enrolled in 17 different residency-training programs as per record of the two teaching hospitals as described below. No residents were excluded from the study.

### Settings

The study was conducted at KKUH, Riyadh and King Abdul-Aziz University Hospital, Riyadh. Both are affiliated to King Saud University, Riyadh, Saudi Arabia. These university hospitals are the largest tertiary care referral teaching hospitals in Riyadh, with a major primary health care facility and various specialized departments, which make them priority targets for many medical students of the country for residency training.

### Data Collection

We distributed the Internet questionnaire, via an email tool, available at a Web site, to the official email list of residents registered in the residency office in the two university hospitals during the period between January 2014 and April 2014. This Web-based survey tool was chosen because it was more reachable by all residents in different departments, in addition to residents who could be rotating outside the two hospitals, where it could be difficult to reach them through conventional paper surveys [13]. There were two reminders that were emailed to nonrespondents one-month apart. Moreover, printed posters encouraging participation with a direct Quick Response code link to the survey website were also provided. Posters were distributed among the hospital departments to motivate the residents to participate in the survey. In addition, we emailed all of the 17 chief residents of different residency-training programs of both university hospitals along with the advertisement poster attached. A text messaging communication service, known as “Tawasol”, which is a Web-based communication service used to communicate with KSU staff members via short text messages, was used to send the official link of the survey to residents. Finally, residents were informally approached individually, asking whether they have participated in the survey or not, and to motivate them to complete it.

### Data Analyses

The data were exported from SurveyMonkey into the Statistical Package for Social Sciences (SPSS), using standardized entry codes. For all tests, statistical significance was set at *P* <.05. Descriptive statistics were used to present means, SD, and percentage. In addition, student’s *t* test, z-proportional test, and chi-square tests were employed to compare group variables between gender and demographic variables. Furthermore, the relationships of resident’s attitudes toward using mobile phones in medical practice were assessed using regression analysis based on gender, specialties, and uses. The model was generated where all the selected variables were converted into binary data (disagree & agree). For multivariable analyses (regression), we constructed a dataset that had complete values for all relevant variables across observations, thereby, discarding the observations that had missing values for any of the variables involved in the regression analysis. The strategy was adopted to maintain comparability between models so that they could be developed from the same denominator. All analyses were conducted using SPSS version 21, 2013 (IBM SPSS, Inc, Chicago, IL).

### Ethical Statement

All participants were informed about the purpose of the study and their electronic consent for participation was taken in the first Web page of the electronic survey. All participants’ data are maintained in a secure fashion by separating participants’ identifiers and associated data. All of the data were analyzed as a total population in a manner that individual privacy was maintained. Institutional Review Board (IRB) approval for this study was taken from the College of Medicine, King Saud University, Riyadh, Saudi Arabia (13/3914/IRB), and the Oregon Health and Science University Research Integrity Office (IRB00010913).

## Results

### Response Rate

Out of the 128 approached residents, 107 responded to the study (83.6%), six of them were excluded due to not answering all or most of the questions. However, surveys with missing answers that were not related to the main outcome variables were included in the analysis. Therefore, a total of 101/133 (75.9%) was analyzed.

### Demographic Information

The mean age of participants was 27.8 years (SD 3.0), ranging from 23 to 38 years old. Most of the participants (59/101, 58.4%) were less than 28 years old. On further comparison, there was no statistically significant difference found between male and female age groups (chi-square test; *P* =.186). Males were higher in number and comprised a total of 63/101 (62.3%) of responded participants. The majority of respondents were in their first year (PGY-1) of residency training (55/101, 54.5%); see (Table 1).

**Table 1:** Demographic feature of the respondents.

Features		n=101	%
**Age (years old)**			
	Mean age (±SD)	27.8 (±3)	
	Below 28	59/101	58.4
	28-32	33/101	32.7
	Above 32	9/101	8.9
**Gender**			
	Male	63/101	62.4
	Female	38/101	37.6
**Residency level**			
	PGY-1	55/101	54.5
	PGY-2	13/101	12.9
	PGY-3	13/101	12.9
	PGY-4	7/101	6.9
	PGY-5	8/101	7.9
	Board eligible	5/101	4.9
**Specialty (top seven only)**			
	Pediatrics	14/101	13.9
	Internal medicine	12/101	11.9
	General surgery	11/101	10.9
	Otorhinolaryngology	11/101	10.9
	Ophthalmology	7/101	6.9
	Family medicine	6/101	5.9
	Obstetrics & gynecology	6/101	5.9

### Mobile Phone Use

Almost all participants reported that they own and use mobile phones (100/101, 99.0%), with mean duration of usage of 5.12 (SD 2.4) years, ranging from 1 to 12 years. The relationship between duration of usage and age was found to follow a linear relationship (duration = 11.6 – 0.234 x age). Although this relationship was statistically significant (ANOVA; *P* =.004), it was not found to be a strong one (*r* =0.282).

The most prevalent language for operating residents’ mobile phones was English (96/100, 96.0%), although Arabic was the native language of most participants (97/101, 96.0%). The operating systems mostly used were iOS from Apple (55/101, 54.5%), followed by Android (54/101, 53.5%), Blackberry (5/101, 5.0%), and Windows Mobile (3/101, 3.0%) in total (Table 2). Further analyses showed that (16/101, 15.8%) of participants were using two different mobile platforms, and only one participant was using three different mobile platforms concurrently. Out of iOS users, 10/45 (22%) were also Android users. All of the three Windows Mobile users reported using other devices at the same time.

**Table 2:** Mobile phone preferences.

		n=101	%
**Mobile phone ownership**			
	Yes	100/101	99.0
	No	1/101	0.9
**Year using a mobile phone**			
	Mean (±SD)	5.18 (±2)	
	Range	1-12 years	
**Operating system used** ^a^			
	iOS	55/101	54.5
	Android	54/101	53.5
	BlackBerry	5/101	4.9
	Windows	3/101	2.9
**Language used on mobile phone** ^b^			
	Arabic	4/101	4.0
	English	96/101	95.0

^a^Total is more than 100%, since more than one choice is allowed.^b^Total is less than 100%, since 1 participant missed answering this question.

### Chatting Apps

**Figure 1:**
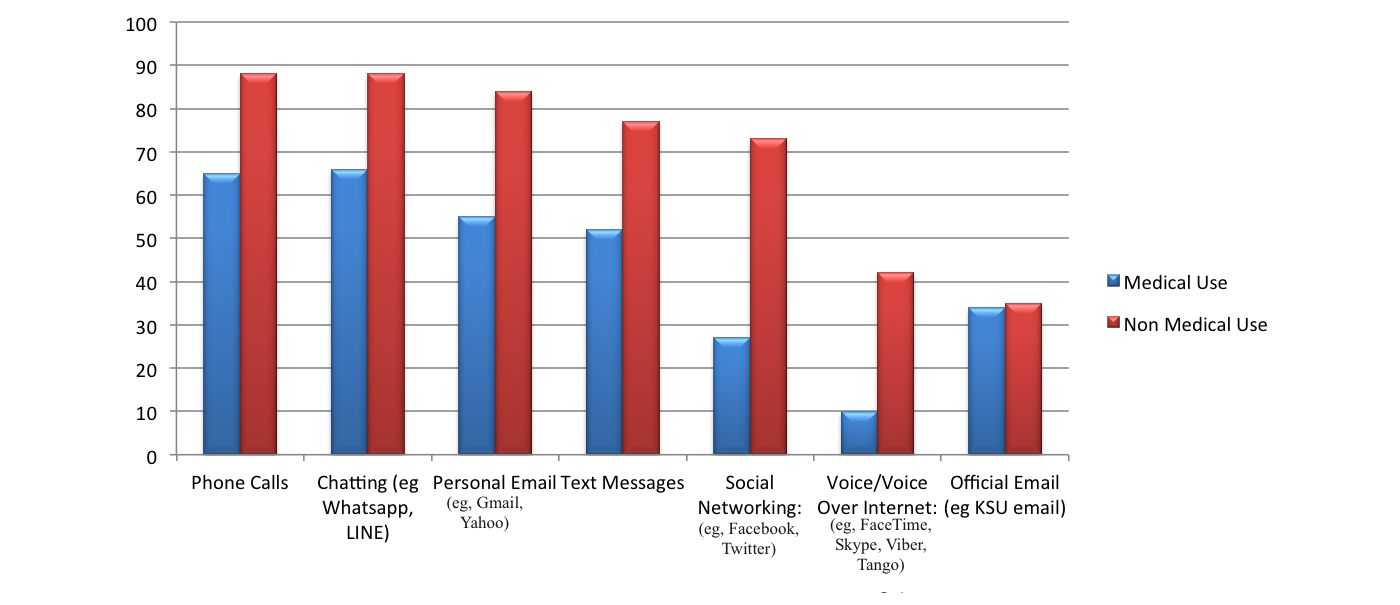
Medical and nonmedical related usage of communication tools/applications (apps). King Saud University: KSU. The numbers on the y axis represent the percentage.

**Figure 2:**
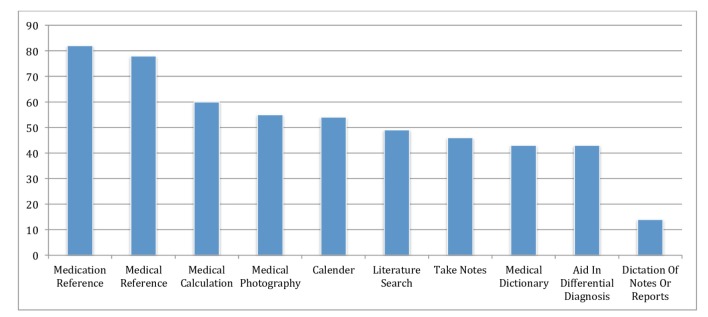
Medical-practice-related noncommunication applications (apps)/tools. The numbers on the y axis represent the percentage.

Chatting apps (88/101, 87.1%) such as WhatsApp and LINE matched traditional phone calls (88/101, 87.1%) as the most often used nonmedical communication tools. Both of these tools, chatting apps (66/101, 65.3%) and phone calls (65/101, 64.4%), were also the highest ranked for practice-related communication, Figure 1 shows this. In general, drug references (83/101, 82.2%); medical references (80/101, 79.2%); and medical calculation (61/101, 60.4%) were the most commonly used noncommunication tools (Figure 2 shows this).

There were four out of five residents (73/92, 79%) that denied awareness of mobile phone medical apps provided by their institutes. There were no significant differences between males and females (chi-square; *P* =.347) or age groups (chi-square; *P* =.326). Most of the participants requested that their institutes should provide access to their patients’ data via mobile version of electronic health records (82/101, 81.2%), mobile drug references (67/101, 66.3%), and medical references (59/101, 58.4%). Almost all of the participants (91/92, 98%) agreed with the idea of integrating medical staff mobile phones with the hospital information system. There were four out of five residents (73/92, 79%) that supported replacing their current hospital pagers with hospital-provided mobile phones.

### Learning to Use Mobile Phones in Medical Practice

When investigating methods of learning how to use mobile phones in medical practice, 84/101 (83.2%) of residents described themselves as self-learners, while 50/101 (49.5%) learned from their peers, while others (27/101, 26.7%) learned from Internet resources such as YouTube and blogs. Only 7/101 (6.9%) were exposed to formal training such as workshops or lectures and seminars about medical uses of mobile phones.

### Reported Useful Uses of Mobile Phones in Medical Practice

Most participants (82/92, 89%) reported that smartphones were useful for staff communication, while 35/92 (38%) reported the communication with patient’s family via mobile phone as harmful, though all the rest of the answers were neutral. In addition, the participants reported that mobile phones were useful in consultation (64/92, 69%), reviewing patient’s lab/radiology results (79/92, 86%), and critical alerts about patients (71/92, 77%).

### Technical Difficulties

**Figure 3:**
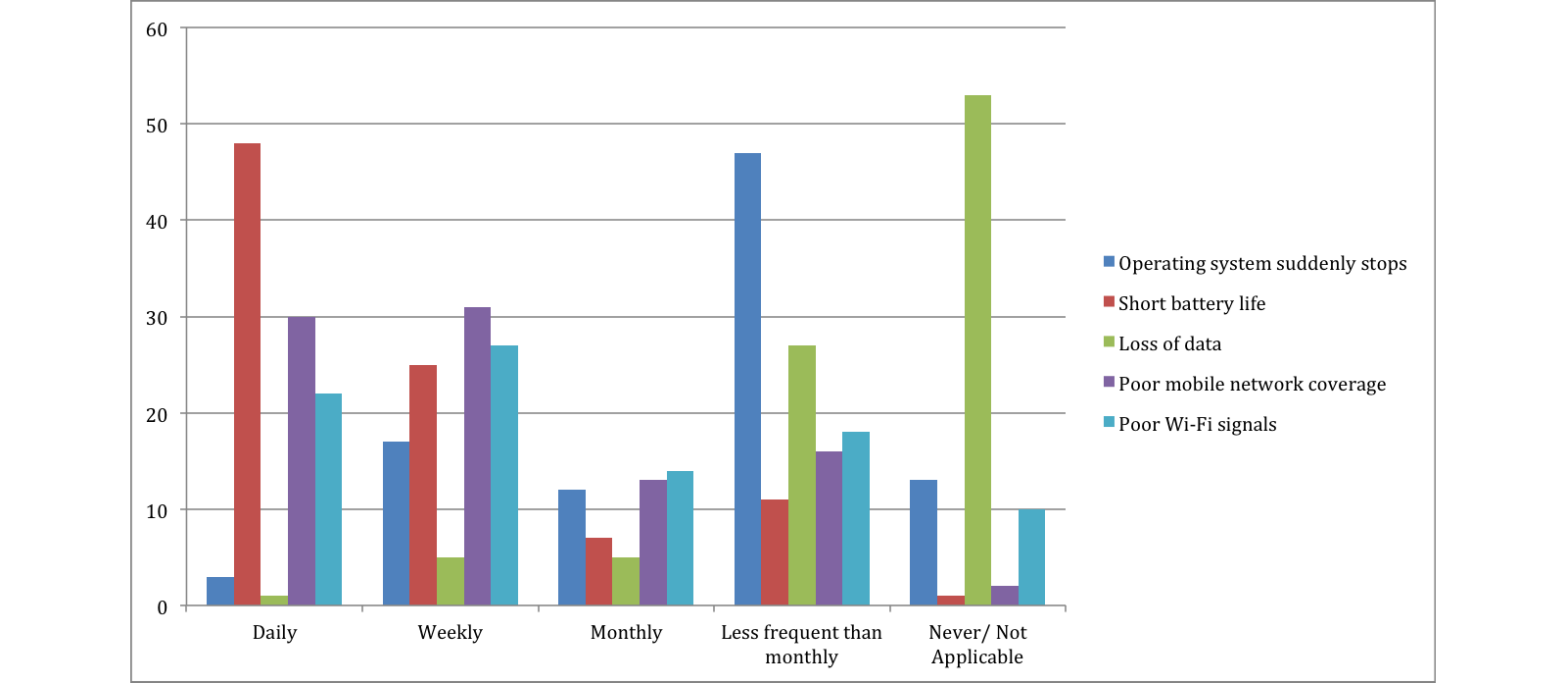
How frequently did you experience these technical problems on your mobile phone? The numbers on the y axis represent the percentage.

**Figure 4:**
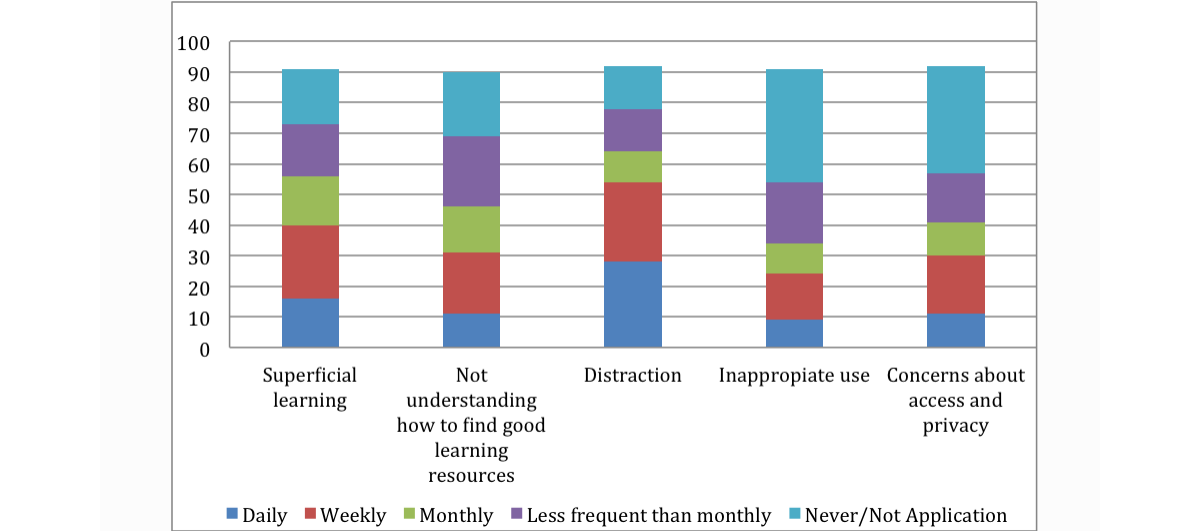
How frequently did you experience these challenges on your mobile phone? The numbers on the y axis represent the percentage.

To find out about the potential technical difficulties residents faced with their mobile phones, they were asked to rate the frequency of difficult situations they faced with their devices. Over half of the participants, (84/92, 91%), reported “short battery life” as a challenge faced on a daily basis (Figure 3 shows this) with 28/92 (30%) reporting that mobile phones were distracting them from their work on a daily basis, and 26/92 (28%) were distracted by smartphones on a weekly basis (Figure 4 shows this).

### Safety of Using Mobile Phones in Medical Practice

**Figure 5:**
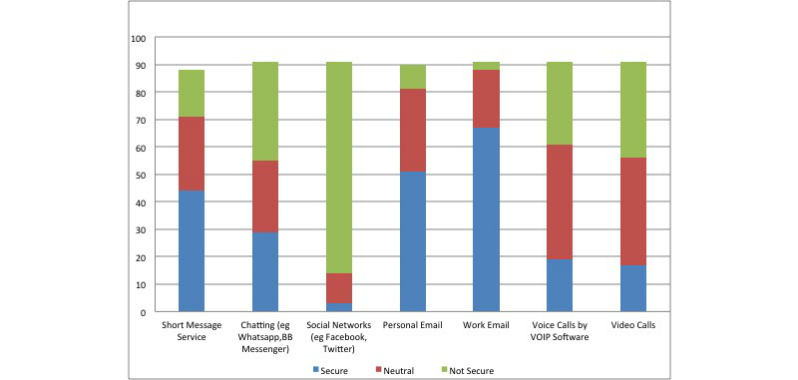
Are these communication application (apps)/tools secure in discussing your patient's details. The numbers on the y axis represent the percentage.

Most of the participants thought that using official email (67/91, 73%) and personal email such as Gmail, Hotmail, and Yahoo (51/92, 55%) were safe and secure in discussing patients’ details. On the other hand, most participants reported social networks, such as Facebook and Twitter (77/92, 84%) were not safe to discuss patients’ data (Figure 5 shows this). Female residents were more conservative in perceiving the safety of mobile chatting apps (χ^2^_2_=8.7, *P* =.012, phi= 0.311) and voice calls apps such as Skype (χ^2^_2_=9.7, *P* =.008, phi= 0.327).

## Discussion

### Principal Findings

Due to the increased global adoption rate of mobile technology in medical practice [8], it is not surprising that the adoption rate found (100/101, 99.0%) in our survey is much higher than previous studies locally and abroad. A comparable study conducted in Riyadh a year prior to this study, but in a much smaller sample size showed an adoption rate of 45.5% among medical residents, with a duration of usage between 5 to 9 years [14]. In the United States, the adoption rate plateaued since 2011 at a low- to mid-80 number in terms of physicians using it for professional purposes [15-17]. In the United Kingdom, a 2012 study quoted a prevalence of 75% [8].

Besides the significance of “how many” residents are using mobile phones, similarly important is “what quality” of apps are being used by these residents. Mobile phone apps for clinical use are a vital aspect for the training residents, who may continue to use such apps for their actual patients’ care. Many studies focused on this issue. For example, Vohralik et al [18] assessed the reliability and validity of a mobile phone app to measure joint range. They concluded that the apps they assessed were both reliable and valid, provided a low-cost method for measuring range of motion, and were easily incorporated into clinical practice. In another study by Man et al [19], they found that a mobile phone app was effective for both increasing confidence in depression treatment and educating physicians. However, they recommended that future studies were still needed to evaluate the effectiveness and impact of mobile phone apps on medical education and postgraduate training [19]. Sampognaro et al [20] reported that mobile devices offered the potential to enhance prerounding efficiency for medical students and residents. They suggested a customizable Evernote-based system for reproduction by interested students [20]. Recently, Jin and Kim [21] described their three-phases evaluation of a “Tool for Healthcare Smartphone Applications”. They concluded that the evaluation tool they developed and tested in their study was an appropriate and widely applicable tool to evaluate health care mobile phone apps to determine if they are reliable and useful [21].

Our study showed a significant negative correlation between age of participants and duration of mobile phone use in their lives. This could be explained by the expectation that younger residents are using mobile phones earlier in their education.

Although Android is the most common mobile platform in the general population [22], Apple’s iPhone iOS was more predominant in medical population, as was found in this study (55/101, 54.5%), which is close to other international surveys (56%) [23].

### Most Commonly Used Apps

Emails are still the primary method of correspondence in health care [24], but are not that common in communication with patients [25,26]. Similarly, communication apps, such as WhatsApp and LINE, usage is gradually wide spreading as much as phone calls as found in this survey, and other literature [27-30]. Our survey showed an increasing prevalence of using mobile nonofficial emails and chatting apps for medical-practice related usage. This is alarming given that 51/90 (56%) of our respondents perceived that it was safe to use their personal nonofficial emails to discuss their patients’ data.

As shown in this study and other previous studies, drug information apps were the most commonly used apps used in clinical settings with a range of 72-100% of residents and physicians [10,14,16,31]. Although our survey did not ask about the use of mobile phones to take clinical photos, other recent studies have reported that the increasing number of physicians, especially dermatologists, capture and store patients’ photos in their mobile phones [30,31].

### Learning Methods

In contrast to most of the other technologies applied in medical practice, the vast majority of mobile phone users, as stated in this study and other studies [32], required only a short time to self-learn how to use their mobile phones for accessing point-of-care medical information at the bedside and engaging in self-directed learning. This highlights their ease of use and potential as a good platform for delivering technology-assisted software. At the same time, we would do well to provide more formal support and training. This would also help residents be more aware of official apps provided or approved by institutions.

### Implications for Practice

A very high percentage of participants (88.9% of male and 90.0% of female participants) strongly agreed that PDAs had improved their performance [14].

The use of handheld computers has improved patient documentation through more complete recording, fewer documentation errors, and increased efficiency. Handheld computers provided easy access to clinical decision support systems and patient management systems, which improved decision making for patient care. Handheld computers saved time and gave earlier access to recent information. There were also reports that handheld computers enhanced work patterns and efficiency [33]. In another study, Tran et al [26] reported on the personal mobile phones uses among clinicians. They found that personal devices were used to communicate with their medical teams and health care professionals. Participants in that study from four academic teaching hospitals in Toronto, Ontario, reported their understanding of the potential risks associated with communicating confidential health information via their personal mobile phones, but appeared to favor efficiency over privacy issues. From survey responses, 9/23 (39%) of the residents reported using their personal cell phones to email or text patient related information that may have contained patient identifiers. Although some residents in that study were observed using personal mobile phones for nonwork-related activities, personal uses were assessed as infrequent [26]. Likewise, in addition, Wu et al [34] described a hospital setting with a newly implemented communication system with support for physician handover and secure messaging. They found that a majority of their medical trainees (82.8%) and nursing staff (78.3%) agreed that such a system helped to speed up their daily work tasks. Most of them also agreed that the system made them more accountable in their clinical roles [34].

Frequent challenges of mobile phone adoption in medical practice found in our survey were limited-battery-life and low-network-coverage. Other studies have raised the awareness of other obstacles such as small screen size, potentially mistaken data input, viruses, magnetic interference with medical devices, hampering of patient-physician interactions, loss or theft, and breaches of data privacy and security [8].

Mobile phone-caused distraction is defined as any interruption of a hospital clinician’s primary task caused by the internally or externally initiated use of mobile phone [35].

### Conclusions

Mobile phone use among medical residents in various medical specialties has become almost universal in health care settings. Despite some limitations, mobile phones play a weighty role in residents’ day-to-day medical practice and residents use them beyond communication. This should alert academic institutes about proper utilization of these devices in medical training and point-of-care decision making, while at the same time ensuring patient’s confidentiality. This may include, but not limited to: suitable training on the proper utilization of these devices, integration of hospital information systems, enabling trainees to access patient’s data on their mobile phones, accompanied by adoption of comprehensive data confidentiality agreement, policies, and procedures. Looking to the future, medical institutions need to incorporate proper use of mobile phone technology and help maximize its potential in their training strategies.

## Abbreviations

apps: applications
ICUs: intensive care units
IRB: Institutional Review Board
KKUH: King Khalid University Hospital
PDAs: personal digital assistant
PGY-1: first year
SPSS: Statistical Package for Social Sciences
